# A virtual reality eyes disease experience for undergraduate nursing students: A phenomenological study protocol

**DOI:** 10.1016/j.mex.2023.102493

**Published:** 2023-11-21

**Authors:** Mauro Parozzi, Paolo Ferrara, Stefano Mancin, Ana Maria Ciorsac, Stefano Terzoni

**Affiliations:** a Biomedicine and Prevention Department, University of Rome “Tor Vergata”, Rome, Italy; b San Paolo bachelor school of Nursing, University of Milan, San Paolo teaching hospital, ASST Santi Paolo e Carlo, via Ovada, 26 – 20142 Milan, Italy; c IRCCS Humanitas Research Hospital Rozzano, Milan, Italy; d Blue Eye Clinic, Milan, Italy

**Keywords:** Virtual simulation, Virtual reality, Nursing education, Nursing students, Eye diseases

## Abstract

Amongst those responsible for the prevention of ocular diseases, registered nurses certainly play an essential role; however, the literature reports that the awareness/consideration of this type of disease by nurses and nursing students can be improved. It is, therefore, appropriate to carry out educational interventions aimed at increasing consideration/awareness of eye diseases consequences through virtual simulation; moreover, the students' experience during this type of activity still needs to be investigated. Therefore, this study aims to explore the experience of glaucoma and cataract sight virtual simulation among Italian nursing students with a qualitative, multi-centric phenomenological research. Bachelor nursing students from different universities in Italy will explore what it means to live with eye diseases through a virtual reality APP. Colaizzi's approach will be used to assess student experiences, investigated through an interview, a pre-experience questionnaire and side notes on student behavior during the experience and the interviews.

## Graphical abstract

**Figure:**
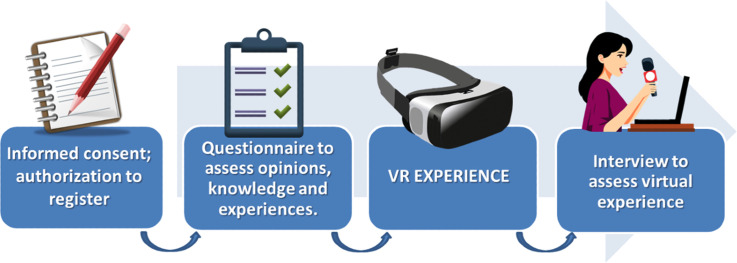

Specifications table

**Table:** 

Subject area:	Medicine and Dentistry
More specific subject area:	Nursing Education
Name of your protocol:	A virtual reality eyes disease experience for undergraduate nursing students: a phenomenological study protocol.
Reagents/tools:	NK 3D VR Glasses for smartphone + NEI VR (See What I See) app.
Experimental design:	Qualitative, multi-centric Phenomenological research. Bachelor nursing students from different universities in Italy will experience eye diseases through a virtual reality APP. Colaizzi's approach will be used to assess student experiences, investigated through an interview, a pre-experience questionnaire and side notes on student behavior during the experience and the interviews.
Trial registration:	N.A.
Ethics:	Following the rules of the ethical committee, the study does not need approval as it falls within the usual training practices without interventions on human beings outside the teaching autonomy provided by law. The training method used in this work will be submitted for approval by the university's campus teaching direction. Participation in the study will be voluntary. Each student intending to participate will be appropriately informed by the researcher and through an appropriate written modular. Students willing to participate to the study will give their informed consent and agree to be recorded during the interview.
Value of the Protocol:	- By the best of our knowledge, in scientific literature, there are no virtual reality experiences on eye disorders carried out by nursing students. - This study will be carried out with readily available materials; this will enable other researchers to replicate the study in different countries around the world. - This is one of the first's Italian studies about the experience of virtual reality in nursing education.

## Description of protocol

### Background

The World Report on Vision by the World Health Organization [1] estimates that globally, at least 2.2 billion people have a visual impairment that could have been prevented in at least 1 billion of these cases.

In Italy, it is estimated that more than 3 million people suffer from vision-threatening conditions, and even more are at risk as these diseases are bound to increase with age and comorbidities [2]. For glaucoma alone, an estimated one million people in Italy are affected by this condition (64 million globally [1]), but half of them are unaware of it [3]. These sight-threatening conditions tend to be asymptomatic in their early stages and can cause enormous social and personal costs; it is clear how prevention in the public health arena may play a crucial role in reducing their incidence and better manage their long term consequences [2].

Among the health professionals in Italy who are deputed to prevention, the nurse certainly has a relevant role; however, globally, the literature would seem to indicate that the knowledge and consideration of eye diseases importance by nurses and nursing students is limited [4], [5], [6], [7].

In fact, upon an initial check by the authors on the curricula published by the Bachelor of Science in Nursing programs of Italian universities, out of 97 universities present, only a minimal number present a program that includes training on ocular pathologies.

Therefore, it makes sense to structure educational interventions that can focus the attention of future nurses on this kind of diseases as well [5] to sentitize them toward how difficult it can be for patients to live with eye diseases. Educational interventions aiming at sensitizing students towards this type of disease have already been tested in the literature, both with physical devices that reproduced particular ocular disease states [8] and with virtual reality technologies [9]; specifically, some virtual reality applications can be downloaded for free and used for the intended purpose, including NEI VR (See What I See), an application created by the National Eye Institute that, as with other experiences in the literature, allows immersion in virtual reality with 360° videos [10]. The experience of nursing students in using this APP for an educational intervention has not yet been investigated.

### AIM

This study aims to explore the perception of students experiencing first-hand virtual simulation of glaucoma and cataracts in an Italian nursing school setting.

### Methods

#### Eligibility

Inclusion Criteria: Bachelor nursing students of any academic year (including repeat students) belonging to one of the universities where the study has been approved.

Exclusion Criteria: Students with a prior personal history of cataracts or glaucoma.

#### Sampling

The convenience sample will consist of students who will voluntarily choose to participate at the study; students will receive an email to their academic email address inviting them to participate in the study.

#### Timing and settings

The study will be coordinated by the “San Paolo” Bachelor School of Nursing (university of Milan); a formal request for participation in the study will be sent to several teaching campuses of different Italian Universities after the protocol will be published.

The study will begin in January 2024 and will end with data saturation, no later than July 2024; the study coordinators will evaluate data saturation.

#### Ethical considerations

Following the rules of the actual ethical committee, the study does not need approval as it falls within the usual training practices without interventions on human beings outside the teaching autonomy provided by law. The training method used in this work will be approved, in any case, by the university's campus teaching direction. Participation in the study will be voluntary. Each student intending to participate will be appropriately informed by the researcher and through an appropriate written consent. They will have to provide their signature on the study informed consent on the study and the forms concerning the authorization to record the interview.

#### The experience

After receiving all the information regarding how the experience will unfold and what will happen during the simulation, the students will be immersed in a virtual reality simulation where they'll deal with two different experiences of “eye disease view.”

In the first experience, the students will see through the eyes of a glaucoma patient standing at a bus stop at night; they will see some buses stopped and others moving as they approach without stopping at the bus stop.

In the second experience, the students will see through the eyes of a patient with cataracts, sitting in the passenger seat while a friend next to him is driving on a lightly trafficked road in a suburban setting.

Every experience will begin with 30 s of healthy view, after which the researchers will activate the "eye disease view." The researchers will alert the students a few seconds before the view change.

The experience will be visual only; the application's audio, which contains an English-language explanation of the displayed pathologies, will be switched off. The researchers will give the information contained in the audio at the end of each experience in Italian language.

#### Procedure

After obtaining appropriate authorizations, students will fill out an initial questionnaire designed to investigate their prior knowledge, opinions, and experiences with diseases/disorders in the field of eye care.

Next, enrolled students will sit, one at a time, in a swivel chair in the center of a room (so that they will not risk touching anything by moving). Educators will give strict instructions; they will be allowed to move their heads in any direction and be able to turn 360° with their chairs but not to stand up for any reason. Similarly, students will be told to communicate to the educators if they experience any physically unpleasant sensations during the virtual experience. In this way, students will be able to observe a 360° virtual reality by being able to move their faces in every direction.

Researchers will then help the students to wear the *VR head mount device,* the sound of the app will switch off and the experience will start.

After 30 s of the "normal" view, the “eye disease view” will be activated, through which students will observe the same everyday scenes with the eyes of a glaucoma patient (for the setting at the bus stop) or cataract patient (for the setting in the car) for additional 30 s.

During the 3d experience, researchers will not communicate with students in any way except to alert them to the change in view; researchers will also note students' movements, externalizations and reactions during the experience. Researchers will only intervene if the student makes dangerous movements or reports physical discomfort during the experience; in such cases, the experience will end immediately.

After the experience is over, it will be evaluated through semi-structured interviews, previously subjected to content validity; the interviews will be recorded, then transcribed and analyzed according to the Colaizzi approach [11].

#### Data management

Data will be handled according to Italian privacy law and GDPR, with pseudoanonymisation of data. Questionnaires will be collected online in a restricted access cloud of the university of Milan. Side notes reported by researchers during the experience and the interview will be collected in separate files and then saved on the same cloud. Interviews will be audio-recorded to allow researchers the opportunity to transcribe them; once transcribed, they will be deleted. The transcripts will be stored on the cloud of the university of Milan. In order to be able to match questionnaires, side notes, and interviews, students will be identified through alphanumeric codes so as to guarantee anonymity.

#### Data analysis

Data analisys will be performed with nVivo software by two Nursing Ph.Ds researcher. with more than 15 years of teaching experience in the nursing university environment.

#### Bracketing

Researchers will deal with bracketing using Chan's four strategies [12].

### Results

We expect to find a generally positive attitude toward virtual reality and a positive perception of firsthand experience of eyes diseases.

## CRediT authorship contribution statement

**Mauro Parozzi:** Conceptualization, Methodology, Supervision, Resources. **Paolo Ferrara:** Methodology, Writing – original draft. **Stefano Mancin:** Conceptualization, Writing – review & editing. **Ana Maria Ciorsac:** Resources, Supervision. **Stefano Terzoni:** Supervision, Writing – review & editing.

## Declaration of Competing Interest

The authors declare that they have no known competing financial interests or personal relationships that could have appeared to influence the work reported in this paper.

## Data Availability

No data was used for the research described in the article.
